# A hypothetical correlation between hyaluronic acid gel and development of cutaneous metaplastic synovial cyst

**DOI:** 10.1186/1746-160X-6-13

**Published:** 2010-07-15

**Authors:** Francesco Inchingolo, Marco Tatullo, Fabio M Abenavoli, Massimo Marrelli, Alessio D Inchingolo, Andrea Servili, Angelo M Inchingolo, Gianna Dipalma

**Affiliations:** 1 Department of Dental Sciences and Surgery, General Hospital, Bari, Italy; 2 Department of Medical Biochemistry, Medical Biology and Physics, General Hospital, Bari, Italy; 3 Department of "Head and Neck Diseases", Hospital "Fatebenefratelli", Rome, Italy; 4 Department of Maxillofacial Surgery, Calabrodental, Crotone, Italy; 5 Department of Dental Sciences and Surgery, General Hospital, Bari, Italy; 6 Department of Maxillofacial Surgery, General Hospital, Bari, Italy; 7 Department of Surgical, Reconstructive and Diagnostic Sciences, General Hospital, Milano, Italy; 8 Department of Dental Sciences and Surgery, General Hospital, Bari, Italy

## Abstract

Thousands of patients receive hyaluronic acid filler injections, and the effects are generally considered acceptable. The acid rarely causes cutaneous reactions, which are only occasionally reported in the literature.

The aim of the present work is to analyze a clinical case that has never been reported in the literature to our knowledge. This case is of a 26-year-old woman who presented with a cyst in the infrazygomatic region that was injected with non-animal stabilized hyaluronic acid at another centre a few months ago.

Consequently, we made an external incision to remove the neoplasm: histological examination of the capsule revealed it to be a cutaneous metaplastic synovial cyst.

## Introduction

Thousands of patients receive hyaluronic acid filler injections, the effects of which are generally considered acceptable. However, in all cases, the product is regarded as resorbable, and the risk of either local or systemic toxic reactions is reduced [1,2].

Hyaluronic acid should be administered with caution, like any type of commonly employed dermal fillers, but the injection technique can be easily performed after a brief learning course. The acid rarely causes cutaneous reactions, which are reported only occasionally in the literature.

## Case Report

**Figure 1:**
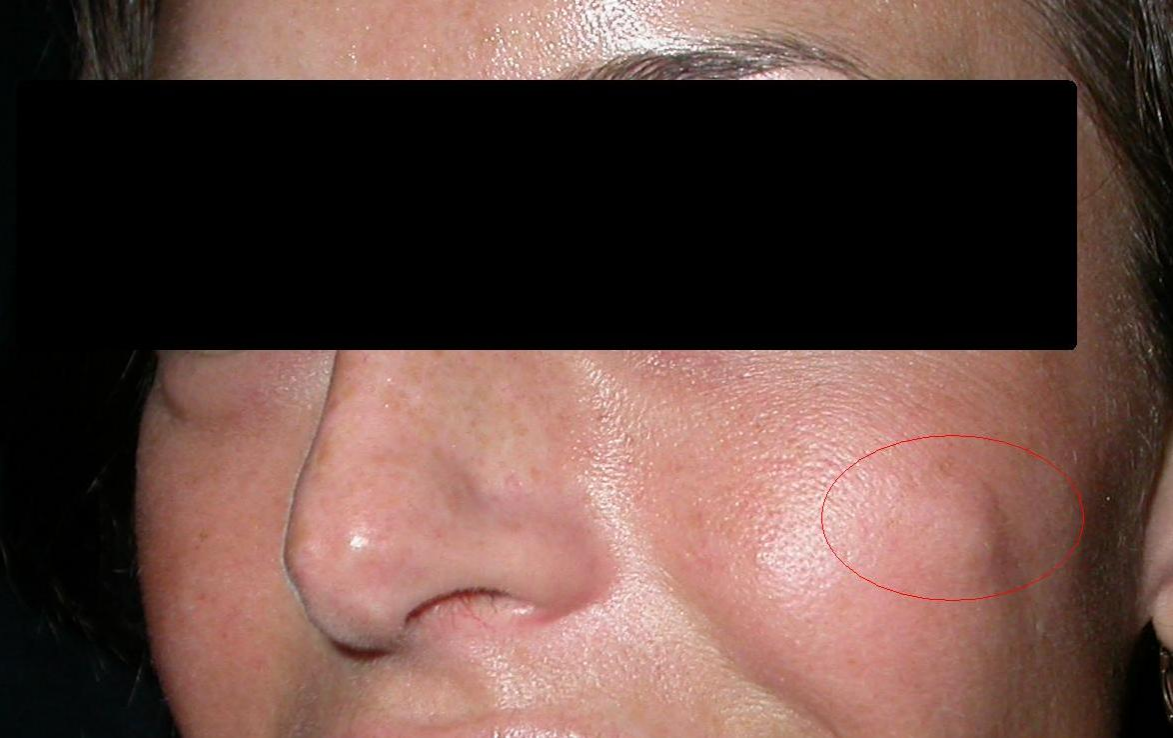
**Preoperative appearance of the neoplastic nodule**.

The aim of the present work is to analyze a clinical case that has never been reported in the literature to our knowledge. This case is of a 26-year-old woman who presented with a cyst in the infrazygomatic region (FIG. 1) that was injected with non-animal stabilized hyaluronic acid (NASHA) at another centre a few months ago. The patient did not have a medical history of cystic formations in that specific region before NASHA injection. However, the cyst tended to increase in volume without any painful sensation or appearance of other symptoms. We performed an MRI that revealed the presence of a cyst that strongly adhered to the bone surface. Consequently, we made an external incision to remove the neoplasm.

In the subcutis, we identified a cyst that we removed with some difficulty because it tenaciously adhered to the underlying bone surface.

**Figure 2:**
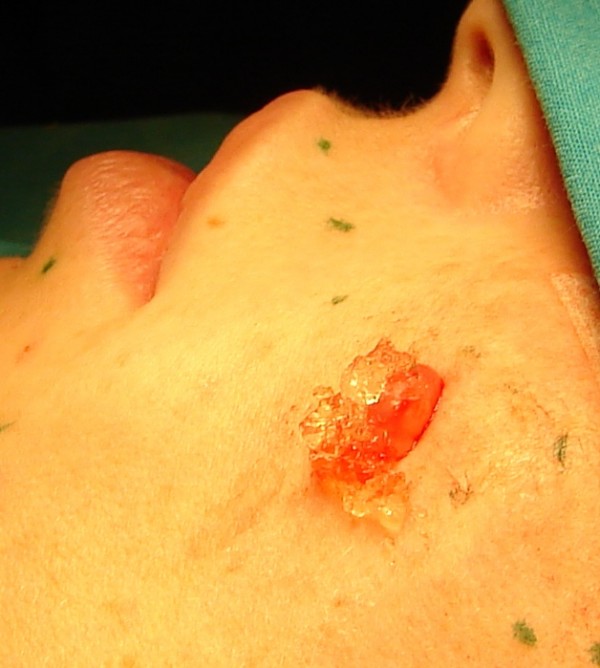
**The cystic lesion filled with a gelatinous material was removed**.

**Figure 3:**
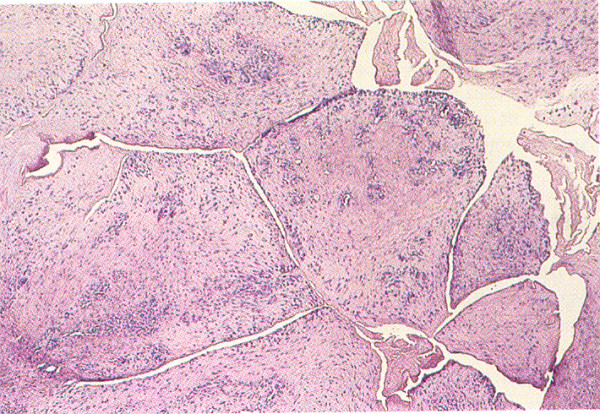
**Results of the histological examination of the cystic capsule**.

After opening the cyst, we observed a thick gelatinous fluid similar to hyaluronic acid (FIG. 2), and histological examination of the capsule revealed the cyst to be a cutaneous metaplastic synovial cyst (FIG. 3).

Cutaneous metaplastic synovial cyst is a rare cystic tumor that typically presents as a solitary tender subcutaneous nodule. Histological examination revealed a cystic structure with villous-like projections and a lining that resembled hyperplastic synovium.

## Discussion

Injection-site reactions result from acute inflammatory reactions in response to tissue damage after drug injection. They are generally short-term and symptoms typically include swelling, pain, tenderness, and bruising; these symptoms can be observed in most patients treated with injectable hyaluronic acid derivatives [3]. Besides, the overall incidence of long-term adverse reactions secondary to injection of hyaluronic acid skin fillers is believed to be low, and the vast majority of them represent a foreign body-related chronic inflammatory reaction.

The cause of formation of cutaneous metaplastic synovial cysts remains unclear, but previous trauma usually precedes its onset. Synovial-like metaplasia associated with prostheses and breast implant capsules has also been reported and may occur in postsurgical cutaneous scars, which are unrelated to prostheses or implants [4-6].

The cyst in the zygomatic region is the element of a seemingly obvious relationship: its formation was attributable to local injection of hyaluronic acid and not to previous local trauma.

Further studies are necessary to determine the underlying mechanism and the causes of this cyst formation.

## Conclusions

If confirmed by other colleagues, it might be interesting to consider the development of cutaneous metaplastic synovial cysts as a possible side effect of the use of hyaluronic acid fillers for increasing the volume of certain regions of the face.

## Consent Statement

Written informed consent was obtained from the patient for publication of this case report and accompanying images. A copy of the written consent is available for review by the Editor-in-Chief of this journal.

## Competing interests

The authors declare that they have no competing interests.

## Authors' contributions

FI: participated in the surgical treatment and in the follow-up of this patient, MT: drafted the manuscript and reviewed the literature, FMA: participated in the surgical treatment and in the follow-up of this patient, MM: participated in the design of this case study and in the follow-up of this patient, ADI: revised the literature sources, AS: participated in the surgical treatment and in the follow-up of this patient, AMI: documented this case report with digital pictures, GD: participated in the follow-up of this patient. All the authors read and approved the final manuscript.
